# Endocrinologic Consequences of Pediatric Posterior Fossa Tumours

**DOI:** 10.4274/jcrpe.2135

**Published:** 2015-12-03

**Authors:** Abdullah Bereket

**Affiliations:** 1 Marmara University Faculty of Medicine, Division of Pediatric Endocrinology, İstanbul, Turkey

**Keywords:** Posterior fossa tumours, children, endocrinologic problems

## Abstract

Intracranial tumors are the second most frequent malignancies in children and posterior fossa is a common location for these neoplasias during childhood. Recent advances in surgical techniques, radiotherapy and chemotherapy resulted in dramatic increase in the survival rates of these children, however they are still source of a significant morbidity and mortality. Endocrinological complications and late sequelae of childhood posterior fossa tumours are common among the survivors of these tumours and include growth retardation, hypothyroidism, pubertal disorders, gonadal dysfunction and osteopenia. These complications have significant impact on the quality of life of the survivors of childhood posterior fossa tumours. In this paper, the frequency, etiology, and management of these complications will be reviewed.

## INTRODUCTION

Primary malignant central nervous system (CNS) tumors are the second most common malignancies in children and are the most common pediatric solid organ tumor (1). The most common tumour types locate in the posterior fossa are cerebellar astrocytomas, medulloblastomas and brain stem gliomas. Although, advances in surgical techniques, new modalities of radiotherapy (RT), and chemotherapy (CT) have greatly improved the survival rates in children with posterior fossa tumours and CNS tumors in general, mortality and morbidity associated with these disorders remain significant.

In most patients with posterior fossa tumors, a combined modality approach that includes surgery, radiation therapy and CT is used in the treatment. Long-term survival is now achieved in majority of the patients, but each component of therapy have a potential to cause late complications that have a profound effect on quality of life in survivors. Endocrine-reproductive problems are among the most common late sequelae in survivors of childhood cancers in general, and in posterior fossa tumors (2). These problems often result in significant morbidity including poor growth, thyroid dysfunction, pubertal delay, gonadal toxicity/infertility, precocious puberty, adrenal insufficency, and osteopenia. Understanding how to improve the prevention, recognition, and treatment of endocrinopathies will improve the well-being of these patients. Children who are at risk for endocrine complications are especially those who were treated with RT and/or high doses of alkylating agents, such as cyclophosphamide, ifosfamide, and busulfan (3). Tumour-induced damage also plays some role in these complications, as shown in a study, in which 15/32 (47%) of patients with posterior fossa tumors had evidence of endocrine problems prior to RT (4).

Medulloblastomas are the most extensively studied posterior fossa tumours in regard to endocrine late consequences. Medulloblastomas are treated with external beam RT to the craniospinal axis, in addition to a boost to the tumor site, after surgical excision of the tumor (5). Contemporary radiation doses differ according to risk group. Average risk patients receive the whole brain and spine treatment with the doses typically around 23.4 Gy, with posterior fossa boost of 30.6 Gy to total dose of 54 Gy. For advanced-stage disease, 36 Gy is given to the whole brain and spine, with a posterior fossa boost of 18 Gy to a total dose of 54 Gy. Severity of the hormonal deficiencies following RT is dose dependent. Low radiation doses (<30 Gy) may cause isolated growth hormone (GH) deficiency in roughly 30% of the patients, while with higher radiation doses of 30-50 Gy, the frequency of GH deficiency can reach 50-100% and long-term gonadotropin, thyroid-stimulating hormone (TSH) and adrenocorticotrophic hormone (ACTH) deficiencies occur in 20-30, 3-9 and 3-6% of the patients, respectively. Precocious puberty can be seen after radiation doses of <30 Gy in girls only, and in both sexes equally with a higher radiation dose (30-50 Gy). Hyperprolactinaemia, due to hypothalamic damage is mostly seen in young women following high dose cranial irradiation but is usually subclinical (6).

New studies focuse on investigating the use of reduced posterior fossa high dose volume boost in order to prevent normal brain tissue from excess radiation exposure. Contemporaray, techniques, such as intensity modulated RT and proton radiation therapy, are under evaluation to limit radiation exposure to normal tissues (7). Although higher doses of RT are associated with better tumor control (5), irradiation of the craniospinal axis in children increase the risk of poor skeletal growth, hypothyroidism, adrenal insufficiency, and hypogonadism, all of which could be minimized with lower doses of radiation and/or newer modalities. In a study comparing reduced dose (18 Gy) with conventional dose (CD) (23 to 39 Gy) of RT, adult heights of the reduced dose group were better than those who received CD. Reduced dose group reached their midparental target heights whereas, CD group remained shorter than their target heights. Of other endocrine sequelae, 10 patients of the CD group were hypothyroid, 3 had central adrenal insufficiency, 3 had hypogonadism, and 2 had early puberty. In contrast, in the reduced dose group, only 1 patient had hypothyroidism and another one had precocious puberty. Thus, endocrinologic problems were significantly reduced with 18 Gy craniospinal RT in young children with medulloblastoma (8).

Because of the above mentioned risks and serious complications, the initial management of childhood medulloblastoma include adjuvant CT with decreased doses of RT in average-risk children or substituted CT for RT in the initial management of infants and young children. However, CT itself has unwanted long-term effects especially on the gonads. Depending on the cumulative doses, cyclophosphamide, Ifosfamide, meclorethamine, procarbazine, busulfan, melphalan and cisplatin are associated with gonadotoxicity in both sexes (9).

## Endocrinologic-Hormonal Complications of Posterior Fossa Tumours

### Growth Retardation and Short Stature

Growth velocity and adult height of pediatric posterior fossa tumour survivors may be negatively influenced by both endocrine and non-endocrine factors. Non-endocrine factors are mainly radiation-induced direct damage to the vertebral growth plates. Spinal irradiation damages both the epiphyseal plates and the bony matrix. Children treated with spinal irradiation often have stunted spinal growth, which becomes more prominent during adolescence (10). Besides this, in the year following diagnosis of medulloblastoma, suboptimal growth may result from ill health and anorexia, although an associated transient GH deficiency is known to occur during or immediately after cranial irradiation. It has been demonstrated that the main reason for suboptimal growth is retardation of spinal growth especially during periods of accelerated growth (11). Brauner et al (12) performed a prospective study of 16 children, aged 1.7-15 year at the time of treatment, who received cranial (31-42 Gy) and spinal radiation for medulloblastoma or ependymoma. Growth of the subjects was compared to that of 11 children given similar doses of cranial radiation alone. After 2 years of follow-up, children treated by cranial and spinal radiation had a mean height of -1.46+/-0.40 standard deviation (SD) below the normal. In contrast, the patients received cranial radiation alone had a mean height of -0.15+/-0.18 SD further supporting the conclusion that suboptimal growth in children with posterior fossa tumors predominantly caused by spinal irradiation.

GH deficiency may also contribute to suboptimal growth in some of these children. In general, GH is the most sensitive hormone to radiation-induced damage, and GH deficiency is therefore, the most common endocrinopathy seen in pediatric cancer survivors following cranial irradiation. GH deficiency develops in a dose- and time-dependent manner with the risk increasing above the doses higher than 18 Gy of radiation and the time interval increases from treatment (13). It has been demonstrated that doses as low as >2.9 Gy cause GH deficiency (14). Younger children are more vulnerable to the efect of radiation-induced GH deficiency as shown in survivors of medulloblastoma. Young age at treatment was a determinant of GH deficiency in adulthood (15).

Early recognition of suboptimal growth in children with posterior fossa tumours are important and requires regular and precise monitoring of both standing height and sitting height measurements. After reviewing the child’s growth curves and treatment-related risk factors, GH provacation tests are indicated in most cases with documented suboptimal growth. Failure in two GH stimulation tests, using two different pharmacologic stimuli, is required for the diagnosis of GH deficiency. In patients who received irradiation, insulin-induced hypoglycemia may be the most sensitive and reliable pharmacologic test of GH status, however because it carries the risk of serious hypoglycemia-related events, it should be performed only in experienced clinics under close surveillance. Insulin-like growth factor-1 (IGF-1) and IGF-binding protein 3 (IGF-BP3), which are commonly used as surrogate markers of GH secretion in children assessed for short stature, are not reliable indicators of GH status following cranial irradiation or documented hypothalamic-pituitary injury due to tumoral expansion (16). In children with posterior fosssa tumors and proven GH deficiency, a comprehensive consultation regarding the benefits and risks of GH therapy should be performed prior to the initiation of therapy. Although several studies have reported no evidence of increased risk of tumor recurrence associated with GH therapy (17), there are data showing a small risk of second neoplasms, particularly solid tumors, in childhood cancer survivors treated with GH (18).

Once the risks and benefits have been carefully discussed, GH therapy can be provided to patients with GH deficiency. Several prognostic factors in regard to adult height was identified. In a report of 183 childhood cancer survivors treated with GH, higher final height was associated with a younger bone age at the time of initiation of GH and higher doses of GH, while a higher dose of spinal irradiation was negatively associated with final height (19).

### Hypothyroidism

Survivors of childhood posterior fossa tumours develop hypothyroidism in varying frequency and the etiology of hypothyroidism depends on the exposure and the dose of radiation. Since both neck and hypothalamopituitary area may have exposed to radiation, the patients can develop primary hypothyroidism, central hypothyroidism or a mixed form of hypothyroidism. The diagnosis of primary hypothyroidism is based on high serum TSH levels and low serum free thyroxine (fT4) values, whereas in central and mixed hypothyroidism low fT4 is accompanied by a normal or modestly elevated TSH. In a study investigating thyroid dysfunction in pediatric posterior fossa tumours, hypothyroidism was found in 12/23 patients in the course of treatment, in 2 patients hormone deficits diagnosed directly after irradiation, in 10 patients, hypothyroidism was observed at the completion of the whole therapy (20).

In survivors of medulloblastoma, both primary and central hypothyroidism has been reported. Primary hypothyroidism is seen more commonly than central hypothyroidism (38% and 19% respectively) (21). Age is an important risk factor as young children are more susceptible to thyroid dysfunction. In a study, all 7 children <5 years developed hypothyroidism, whereas, in children of 5-10 years, and >10 years frequency of hypothyroidism were 60% and 20% respectively. Furthermore, hypothyroidism was shown in 83% who had 23 Gy+CT, 60% who had 36 Gy+CT, and 20% who had 36 Gy without CT indicating that regimens consisting of CT and 2340 cGy craniospinal irradiation followed by a posterior fossa boost for medulloblastoma do not show a reduction of hypothyroidism. However, Ricardi et al (22) have shown that the use of hyperfractionated craniospinal RT in the treatment of childhood posterior fossa primitive neuroectodermal tumours is associated with a lower risk of developing late thyroid dysfunction. Risk factors for hypothyroidism include young age, use of CT and CDs of RT, all of which are associated with a higher incidence of hypothyroidism. Levothyroxine treatment is indicated for patients with hypothyroidism. Dose should be titrated to maintain normal fT4 levels. Serum TSH should be monitored to assess dosing adequacy and medication compliance in cases with primary hypothyroidism.

### Thyroid Neoplasms

Exposure of radiation to neck during craniospinal irradiation in children with posterior fossa tumours pose a risk for both benign and malignant thyroid neoplasms (23). The raised TSH values in association with the radiation damage to the thyroid may be an important factor in carcinogenesis (24). Thus, treatment with T4 to suppress elevated TSH may be appropriate in these patients. RT prior to 10 years of age, and/or total doses of radiation between 20 and 29 Gy appear to confer the greatest risk for the development of thyroid cancer (23). The association between radiation dose and thyroid cancer is curvilinear, with risk increasing at low to moderate doses and decreasing at doses >30 Gy due to cell-killing effect (25). Among patients treated with radiation doses to the thyroid of ≤20 Gy, treatment with alkylating agents appears to increase the risk of thyroid neoplasias (26). The risk of thyroid cancer persists throughout the adult life of at-risk survivors. There are case reports of thyroid carcinoma developed 7-12 years after irradiation for medulloblastoma (27,28). The Children’s Oncology Group currently recommends yearly examination of the thyroid gland via careful palpation. Routine use of screening thyroid ultrasonography in childhood cancer survivors increases detection of small nodules of uncertain clinical significance and may result in unnecessary and excessive invasive procedures. This was illustrated in a study of pediatric Hodgkin lymphoma survivors who underwent routine screening thyroid ultrasonography. Although thyroid nodules were a common finding, only one case of malignant thyroid cancer was detected by ultrasound screening. Another six cases of thyroid cancer developed in the cohort, which were detected after clinical findings prompted further evaluation. All seven patients with thyroid cancer were alive at the time of data analysis (29). Thus, in survivors of posterior fossa tumours, careful palpation and/or thyroid ultrason (US) annually is recommended. In patients who have suspicious nodules (based on ultrasonographic and clinical features), a fine needle aspiration biopsy is required to rule out malignancy. Continous surveillance with US is needed in those with benign cytology.

### Delayed Puberty

Hypogonadotroic hypogonadism or gonadal damage may cause delayed or arrested puberty depending on age in the survivors of childhood posterior fossa tumours. Those who have already reached sexual maturity can also develop treatment-related luteinizing hormone (LH)/follicle-stimulating hormone (FSH) deficiency. Presentation in adul females includes secondary amenorrhea, and in males, loss of libido, erectile dysfunction, and reduced energy.

Delayed puberty is defined by the absence of breast development in girls by 13 years of age and testicular size less than 4 ml in boys by 14 years of age. Absence of progression or completion of puberty that had already started is called arrested puberty. The patients who are treated with radiation doses >30 to 40 Gy to the hypothalamic-pituitary axis are at risk for deficits of LH and FSH called hypogonadotropic hypogonadism (low Serum FSH and LH levels) (30). In addition to hypogonadotropic hypogonadism, survivors of childhood posterior fossa tumours are at risk for primary gonadal dysfunction due to direct damage to the ovaries or testes (in which case serum FSH and LH are elevated and is called Hypergonadotropic hypogonadism). Similar to those in hypothyroidism, some patients with gonadal failure may also have partial gonadotropin deficiency which prevents elevation of gonadotropins and mask gonadal damage. Mesaurement of inhibin B and antimüllerian hormone (AMH) may be helpful in these circumstances. Cuny et al (31) measured plasma inhibin B and in 34 boys and 22 girls to evaluate the roles of hypothalamic-pituitary and spinal irradiations and CT in gonadal dysfunction after treatment for medulloblastoma or posterior fossa ependymoma. Two boys had partial gonadotropin deficiency, one of them was combined with testicular deficiency. Six boys had increased levels of FSH, indicating tubular deficiency, combined with Leydig cell deficiency in 5 of them. Inhibin B levels were <100 ng/mL in 7 boys (6 boys with testicular damage, and one boy with combined deficiencies. Puberty did not progress in 7 girls; 3 had gonadotropin deficiency (one of them also had gonadotropin deficiency), and 4 had increased FSH levels indicative of ovarian damage. Inhibin B and AMH levels were low in the girl with combined deficiency, in the 4 girls with ovarian damage, and in 4 girls with normal clinical-biological ovarian function, including twogirls who underwent ovarian transposition before irradiation. Thus, it appaears that low plasma concentrations of inhibin B and AMH are useful means of detecting primary gonadal deficiency in patients who have no increase in their plasma gonadotropin levels because of radiation-induced gonadotropin deficiency.

Hypogonadotropic hypogonadism in survivors of posterior fossa tumors results from radiation exposure of brain, whereas primary gonadal failure may result from both radiation exposure to gonads and/or damaging effects of CT to the gonads. Ovarian (32) and testicular (33) damage after abdominal irradiation in childhood is known for a long time. Damage to the gonad is influenced by the total radiation dose, fractionation and time sequence of treatment and the sex and age of the patient. Single doses of 6 Gy are probably 100% effective in causing permanent sterility in women of all ages (34). Brown et al (35) have shown that after spinal irradiation of 35 Gy the scattered dose to the ovaries may be as high as 10 Gy, and by the use of skin dose meters they calculated a cumulative dose of approximately 2.4 Gy to the testes. It has been shown that patients who had had CCNU but no spinal RT also had evidence of primary gonadal damage (35).

### Male Patients

The human testis has two primary functions: sperm production and testosterone production. One or both of these functions may be damaged by cancer treatment. Germ cells and Sertoli cells form the seminiferous tubules where spermatogenesis occurs; Leydig cells are responsible for the production of testosterone.

### Germ cell dysfunction:

The following chemotherapeutic agents are associated with impaired spermatogenesis, which is dependent on the cumulative dose (36): Mechlorethamine, Cyclophosphamide, Ifosfamide, Procarbazine, Busulfan, Melphalan (all alkylating agents) and Cisplatin. Alkylating agents used in concert have additive gonadotoxic effects. Earlier studies suggested that younger age at treatment was associated with a lower risk of germ cell loss, however, data in this matter are inconclusive. Sperm analysis is the only definitive test available to determine a survivor’s ability to produce sperm. Although a variety of clinical (e.g., decreased testicular volume) and biochemical findings (e.g., raised plasma concentrations of FSH and reduced plasma concentrations of inhibin-B) have been associated with impaired sperm production in population studies, none is suitable as diagnostic for oligospermia due to poor sensitivity and/or specificity.

### Leydig cell dysfunction:

Leydig cells are susceptible to radiation-induced damage at higher doses than those associated with germ cell dysfunction and the risk is directly related to testicular radiation dose and inversely related to age at treatment (37). The majority of males who receive <20 Gy fractionated radiation to the testes will continue to produce normal amounts of testosterone (37). However, most prepubertal males who receive radiation doses ≥24 Gy to the testis will develop Leydig cell failure. CT alone rarely results in Leydig cell failure, but subclinical Leydig cell dysfunction has been reported more commonly following treatment with alkylating agents (36). Ahmed et al (38) showed in a group of patients with medulloblastoma who received surgery and craniospinal radiation, only those who received CT had evidence of gonadal failure. They concluded that nitrosoureas were mainly responsible for the gonadal damage in these children and procarbazine also contributing to the damage in the three children who received this drug. The authors questioned the necessity of adjuvant CT in view of the limited proved value of adjuvant CT with nitrosoureas in the treatment of medulloblastoma, and recognition of this serious complication of cytotoxic drug therapy. Leydig cell failure will result in delayed puberty if it occurs before pubertal onset, or pubertal arrest if it occurs after the start of puberty. Affected males who have completed normal puberty may present with reduced libido, erectile dysfunction, decreased bone mineral density (BMD), and decreased muscle mass. In Leydig cell failure serum levels of LH is elevated and testosterone levels are low. These patients should be referred to an endocrinologist for initiation of testosterone replacement therapy.

### Female Patients

Ovarian dysfunction may result from treatment with gonadotoxic CT (especially alkylating agents), or radiation-induced damage to the ovaries (31,32,35,39). Due to the interdependence of the sex steroid-producing cells and oocytes within the ovarian follicle, ovarian failure results in impairment of both sex hormone production and fertility. Risk of ovarian dysfunction is directly correlated with cumulative dose and age at exposure. Radiation doses to the ovary exceeding 10 Gy are associated with a very high risk of ovarian failure (40). When ovarian transposition is performed prior to RT, many girls retain ovarian function (41). Furthermore, it has been shown that using magnetic resonance imaging to localise the ovaries for positioning and a modified RT technique using a non-divergent beam edge inferiorly, it is possible to to reduce the ovarian dose by 66% (42). This technique was able to reduce the number of patients receiving <4 Gy to a single ovary from three to six. Irradiation at older age confers a greater risk. Women who have normal ovarian function at the end of treatment with potentially gonadotoxic therapy remain at risk for premature menopause later in life and should be counseled accordingly (43). All patients treated with the gonadotoxic therapies should have periodic screening of LH and FSH measurements, and Tanner staging to monitor pubertal progression. Elevated gonadotropin levels indicate ovarian damage. Patients with ovarian deficiency should be referred to an endocrinologist for initiation of ovarian hormone replacement therapy.

### Precocious Puberty

In survivors of CNS malignancies, or those treated with cranial RT, puberty may progress at a rapid tempo with accelerated skeletal development resulting in short stature (44,45,46,47,48). In a recent study, the prevalance of central precocious puberty was found as 3.2% in children with posterior fossa tumors (45). Younger age at diagnosis, female sex, and increased body mass index (BMI) are among the risk factors associated with precocious puberty (46). Radiation doses to hypothalamo-pituitary area 30-55 Gy is associated with precocious puberty in childhood malignancies, doses higher than that more likely to result in gonadotrophin deficiency (47). It should be noted that children with precocious puberty due to cranial irradiation usually have GH deficiency as well, which may be masked since growth velocity is not slow because of precocious puberty. Survivors of childhood posterior fossa tumours who have early onset and/or a rapid tempo of pubertal development should be referred to an endocrinologist. Assessment of skeletal maturity via standard bone age and measurement of LH, FSH testosterone/estradiol levels should be done. A pelvic ultrasound, which assesses size of the ovaries and uterus, also may be obtained in girls. Since central precocious puberty is associated with accelerated bone age advancement with resultant reduction in final height potential, it may be beneficial to temporally suppress the hypothalamic-pituitary-gonadal axis by using long-acting formulations of gonadotropin releasing hormone agonists. Such therapy may prevent early menarche and further advancement in skeletal maturity, allowing a modest improvement in final height.

### Adrenocorticotrophic Hormone Deficiency

ACTH deficiency is not a frequent endocrinopathy in posterior fossa tumour survivors and if present, it is related to high-dose irradiation >30 Gy to hypothalamo-pituitary area resulting in ACTH deficiency (49,50). Transient ACTH deficiency may also result from prolonged use of pharmacologic doses of glucocorticoids. Patients with ACTH deficiency may present with fatigue, poor weight gain, and/or hypoglycemia. In times of stress or illness, unrecognized ACTH deficiency can be life-threatening. The patients should be screened annually for ACTH deficiency, by obtaining a fasting morning cortisol level at 8 AM. At-risk patients with screening basal cortisol levels <10 mcg/dL (276 nmol/L), or those who are symptomatic, should be referred to an endocrinologist for dynamic testing of adrenal function. Tests for appropriate central regulation of adrenal function include the insulin tolerance test, glucagon stimulation test, metyrapone test, standard-dose ore low dose ACTH stimulation tests. Patients with ACTH deficiency require glucocorticoid replacement therapy (e.g., hydrocortisone, prednisone, or prednisolone) at physiologic doses based on a daily production rate of hydrocortisone of 7 mg/m2 per day. Under stress conditions, such as illness or surgery, glucocorticoid dosing should be increased to three times the normal replacement dose. If the patient is unable to tolerate oral therapy, an intramuscular injection of approximately 50 mg/m2 of hydrocortisone sodium succinate (Solu-Cortef) should be administered. Every patient with ACTH deficiency should wear a medical identification bracelet, indicating the diagnosis of adrenal insufficiency, at all times.

### Low Bone Mineral Density

Dual energy x-ray absorptiometry (DEXA) has traditionally been used to determine BMD. However it is important to emphasize that the results obtained with DEXA in children and adolescents must be interpreted according to age, height, and pubertal stage using normative Z-scores rather than T-scores. Children who have undergone irradiation for posterior fossa tumors have diminished total body and lumbar spine BMD, as compared with those of the general population (51,52,53). In most of these patients, BMD was lower than normal in both the lumbar column and in the femoral neck. However, bone mass loss was higher in the lumbar region than in the femoral neck, due to spinal radiation therapy and to the effect of hormonal deficiencies. Hypogonadism in particular, but also multiple hormonal deficiencies, are associated with lower BMD values. In a study, regional BMD has been measured using DEXA in adults following craniospinal irradiation for medulloblastoma between ages 4 and 19 years, receiving doses of 35-40 Gy to the brain and spinal cord. Failure to achieve a normal BMD and mean reduction at lumbar spine of 12.1% and a mean reduction at femoral neck of 14.3% was observed (52). There was no relationship between reduction in BMD at either site and age at irradiation, time elapsed since irradiation or BMI at time of scanning. Biochemical and endocrine evaluation including calcium, alkaline phosphatase, sex hormones and IGF-1 were normal in all patients. The observation of reduced BMD outside the irradiated area as well suggests that indirect factors may be important in osteopenia seen in these patients. In another study performed in the survivors of posterior fossa tumors, the reduction in BMD was similar within all treatment groups (craniospinal irradiation and CT, only craniospinal irradiation, and only posterior fossa irradiation), suggesting that CT did not play a major role in osteopenia and that localized irradiation may have systemic effects (53). This population often has balance and gait problems, so the risk of falling, coupled with osteopenia, may place them at considerably increased risk of fractures.

In addition to radiation and gonadotropin deficiency, GH deficiency may contribute to low BMD in survivors of posterior fossa tumours. Sedentary lifestyle and suboptimal nutrition, which is often problematic in these group of children may also worsen BMD. Subjects at high risk for low BMD and those who experience fractures should undergo screening with bone density studies at entry into a long-term follow-up program. Calcium and vitamin D supplementation, and regular weight-bearing exercise should be encouraged in these children with borderline or low BMD. Sex hormone and GH replacement therapies should be implemented in those with hormone deficiencies to prevent pathological fractures, thus improving the quality of life.

Peer-review: Internal peer-reviewed, Financial Disclosure: The author declared that this study has received no financial support.

## References

[1] Linabery AM, Ross JA (2008). Trends in childhood cancer incidence in the U.S. (1992-2004). Cancer.

[2] Hudson MM, Ness KK, Gurney JG, Mulrooney DA, Chemaitilly W, Krull KR, Green DM, Armstrong GT, Nottage KA, Jones KE, Sklar CA, Srivastava DK, Robison LL (2013). Clinical ascertainment of health outcomes among adults treated for childhood cancer. JAMA.

[3] Nandagopal R, Laverdière C, Mulrooney D, Hudson MM, Meacham L (2008). Endocrine late effects of childhood cancer therapy: a report from the Children’s Oncology Group. Horm Res.

[4] Merchant TE, Williams T, Smith JM, Rose SR, Danish RK, Burghen GA, Kun LE, Lustig RH (2002). Preirradiation endocrinopathies in pediatric brain tumor patients determined by dynamic tests of endocrine function. Int J Radiat Oncol Biol Phys.

[5] Hughes EN, Shillito J, Sallan SE, Loeffler JS, Cassady JR, Tarbell NJ (1988). Medulloblastoma at the joint center for radiation therapy between 1968 and 1984. The influence of radiation dose on the patterns of failure and survival. Cancer.

[6] Darzy KH, Shalet SM (2009). Hypopituitarism following radiotherapy revisited. Endocr Dev.

[7] St Clair WH, Adams JA, Bues M, Fullerton BC, La Shell S, Kooy HM, Loeffler JS, Tarbell NJ (2004). Advantage of protons compared to conventional X-ray or IMRT in the treatment of a pediatric patient with medulloblastoma. Int J Radiat Oncol Biol Phys.

[8] Xu W, Janss A, Packer RJ, Phillips P, Goldwein J, Moshang T (2004). Endocrine outcome in children with medulloblastoma treated with 18 Gy of craniospinal radiation therapy. Neuro Oncol.

[9] Kenney LB, Cohen LE, Shnorhavorian M, Metzger ML, Lockart B, Hijiya N, Duffey-Lind E, Constine L, Green D, Meacham L (2012). Male reproductive health after childhood, adolescent, and young adult cancers: a report from the Children’s Oncology Group. J Clin Oncol.

[10] Shalet SM, Gibson B, Swindell R, Pearson D (1987). Effect of spinal irradiation on growth. Arch Dis Child.

[11] Broadbent VA, Barnes ND, Wheeler TK (1981). Medulloblastoma in childhood:long term results of treatment. Cancer.

[12] Brauner R, Rappaport R, Prevot C, Czernichow P, Zucker JM, Bataini P, Lemerle J, Sarrazin D, Guyda HJ (1989). A prospective study of the development of growth hormone deficiency in children given cranial irradiation, and its relation to statural growth. J Clin Endocrinol Metab.

[13] Darzy KH (2009). Radiation-induced hypopituitarism after cancer therapy: who, how and when to test. Nat Clin Pract Endocrinol Metab.

[14] Shalet SM, Beardwell CG, Pearson D, Morris-Jones PH (1976). The effect of varying doses of cerebral irradiation on growth hormone production in childhood. Clin Endocrinol (Oxf).

[15] Heikens J, Michiels EM, Behrendt H, Endert E, Bakker PJ, Fliers E (1998). Long-term neuro-endocrine sequelae after treatment for childhood medulloblastoma. Eur J Cancer.

[16] Weinzimer SA, Homan SA, Ferry RJ, Moshang T (1999). Serum IGF-I and IGFBP-3 concentrations do not accurately predict growth hormone deficiency in children with brain tumours. Clin Endocrinol (Oxf).

[17] Packer RJ, Boyett JM, Janss AJ, Stavrou T, Kun L, Wisoff J, Russo C, Geyer R, Phillips P, Kieran M, Greenberg M, Goldman S, Hyder D, Heideman R, Jones-Wallace D, August GP, Smith SH, Moshang T (2001). Growth hormone replacement therapy in children with medulloblastoma: use and effect on tumor control. J Clin Oncol.

[18] Ergun-Longmire B, Mertens AC, Mitby P, Qin J, Heller G, Shi W, Yasui Y, Robison LL, Sklar CA (2006). Growth hormone treatment and risk of second neoplasms in the childhood cancer survivor. J Endocrinol Metab.

[19] Brownstein CM, Mertens AC, Mitby PA, Stovall M, Qin J, Heller G, Robison LL, Sklar CA (2004). Factors that affect final height and change in height standard deviation scores in survivors of childhood cancer treated with growth hormone: a report from the childhood cancer survivor study. J Clin Endocrinol Metab.

[20] Sobol G, Musioc K, Kalina M, Kalina-Faska B, Mizia-Malarz A, Ficek K, Woc H, Macecka-Tendera E (2012). The evaluation of function and the ultrasonographic picture of thyroid inchildren treated for medulloblastoma. Childs Nerv Syst.

[21] Paulino AC (2002). Hypothyroidism in children with medulloblastoma: a comparison of 3600 and 2340 cGy craniospinal radiotherapy. Int J Radiat Oncol Biol Phys.

[22] Ricardi U, Corrias A, Einaudi S, Genitori L, Sandri A, di Montezemolo LC, Besenzon L, Madon E, Urgesi A (2001). Thyroid dysfunction as a late effect in childhood medulloblastoma: a comparisonof hyperfractionated versus conventionally fractionated craniospinal radiotherapy. Int J Radiat Oncol Biol Phys.

[23] Bhatti P, Veiga LH, Ronckers CM, Sigurdson AJ, Stovall M, Smith SA, Weathers R, Leisenring W, Mertens AC, Hammond S, Friedman DL, Neglia JP, Meadows AT, Donaldson SS, Sklar CA, Robison LL, Inskip PD (2010). Risk of second primary thyroid cancer after radiotherapy for a childhood cancer in a large cohort study: an update from the childhood cancer survivor study. Radiat Res.

[24] Conard RA, Rall JE, Sutow WW (1966). Thyroid nodules as a late sequela of radioactive fallout, In a Marshall Island population exposed in 1954. N Engl J Med.

[25] Sigurdson AJ, Ronckers CM, Mertens AC, Stovall M, Smith SA, Liu Y, Berkow RL, Hammond S, Neglia JP, Meadows AT, Sklar CA, Robison LL, Inskip PD (2005). Primary thyroid cancer after a first tumour in childhood (the Childhood Cancer Survivor Study): a nested case-control study. Lancet.

[26] Veiga LH, Bhatti P, Ronckers CM, Sigurdson AJ, Stovall M, Smith SA, Weathers R, Leisenring W, Mertens AC, Hammond S, Neglia JP, Meadows AT, Donaldson SS, Sklar CA, Friedman DL, Robison LL, Inskip PD (2012). Chemotherapy and thyroid cancer risk: a report from the childhood cancer survivor study. Cancer Epidemiol Biomarkers Prev.

[27] Raventos A, Duszynski DO (1963). Thyroid cancer following irradiation for medulloblastoma. AJR.

[28] Andrew DS, Kerr IF (1965). Carcinoma of thyroid following irradiation for medulloblastoma. Clin Radiol.

[29] Metzger ML, Howard SC, Hudson MM, Gow KW, Li CS, Krasin MJ, Merchant T, Kun L, Shelso J, Pui CH, Shochat SJ, McCarville MB (2006). Natural history of thyroid nodules in survivors of pediatric Hodgkin lymphoma. Pediatr Blood Cancer.

[30] Sklar CA, Constine LS (1995). Chronic neuroendocrinological sequelae of radiation therapy. Int J Radiat Oncol Biol Phys.

[31] Cuny A, Trivin C, Brailly-Tabard S, Adan L, Zerah M, Sainte-Rose C, Alapetite C, Brugières L, Habrand JL, Doz F, Brauner R (2011). Inhibin B and anti-Müllerian hormone as markers of gonadal function after treatment for medulloblastoma or posterior fossa ependymoma during childhood. J Pediatr.

[32] Shalet SM, Beardwell CG, Jones PH, Pearson D, Orrell DH (1976). Ovarian failure following abdominal irradiation in childhood. Br J Cancer.

[33] Shalet SM, Beardwell CG, Jacobs HS, Pearson D (1978). Testicular function following irradiation of the human prepubertal testis. Clin Endocrinol (Oxf).

[34] Ash P (1980). The influence of radiation on fertility in man. Br J Radiol.

[35] Brown IH, Lee TJ, Eden OB (1983). Growth and endocrine function after treatment for medulloblastoma. Arch Dis Child.

[36] Kenney LB, Cohen LE, Shnorhavorian M, Metzger ML, Lockart B, Hijiya N, Duffey-Lind E, Constine L, Green D, Meacham L (2012). Male reproductive health after childhood, adolescent, and young adult cancers: a report from the Children’s Oncology Group. J Clin Oncol.

[37] Sklar C (1999). Reproductive physiology and treatment-related loss of sex hormone production. Med Pediatr Oncol.

[38] Ahmed SR, Shalet SM, Campbell RH, Deakin DP (1983). Primary gonadal damage following treatment of brain tumors in childhood. J Pediatr.

[39] Clayton PE, Shalet SM, Price DA, Jones PH (1989). Ovarian function following chemotherapy for childhood brain tumours. Med Pediatr Oncol.

[40] Green DM, Whitton JA, Stovall M, Mertens AC, Donaldson SS, Ruymann FB, Pendergrass TW, Robison LL (2002). Pregnancy outcome of female survivors of childhood cancer: a report from the Childhood Cancer Survivor Study. Am J Obstet Gynecol.

[41] Barahmeh S, Al masri M, Badran O, Masarweh M, El-Ghanem M, Jaradat I, Lataifeh I (2013). Ovarian transposition before pelvic irradiation: indications and functional outcome. J Obstet Gynaecol Res.

[42] Harden SV, Twyman N, Lomas DJ, Williams D, Burnet NG, Williams MV (2003). A method for reducing ovarian doses in whole neuro-axis irradiation for medulloblastoma. Radiother Oncol.

[43] Chemaitilly W, Mertens AC, Mitby P, Whitton J, Stovall M, Yasui Y, Robison LL, Sklar CA (2006). Acute ovarian failure in the childhood cancer survivor study. J Clin Endocrinol Metab.

[44] Armstrong GT, Chow EJ, Sklar CA (2009). Alterations in pubertal timing following therapy for childhood malignancies. Endocr Dev.

[45] Chemaitilly W, Merchant TE, Li Z, Barnes N, Armstrong GT, Ness KK, Pui CH, Kun LE, Robison LL, Hudson MM, Sklar CA, Gajjar A (2015 Oct 14). Central Precocious Puberty following the Diagnosis and Treatment of Paediatric Cancer and Central Nervous System Tumours: Presentation and Long-term Outcomes. Clin Endocrinol (Oxf).

[46] Oberfield SE, Soranno D, Nirenberg A, Heller G, Allen JC, David R, Levine LS, Sklar CA (1996). Age at onset of puberty following high-dose central nervous system radiation therapy. Arch Pediatr Adolesc Med.

[47] Müller J (2002). Disturbance of pubertal development after cancer treatment. Best Pract Res Clin Endocrinol Metab.

[48] Josan VA, Timms CD, Rickert C, Wallace D (2007). Cerebellar astrocytoma presentingwith precocious puberty in a girl. Case report. J Neurosurg.

[49] Rose SR, Danish RK, Kearney NS, Schreiber RE, Lustig RH, Burghen GA, Hudson MM (2005). ACTH deficiency in childhood cancer survivors. Pediatr Blood Cancer.

[50] Patterson BC, Truxillo L, Wasilewski-Masker K, Mertens AC, Meacham LR (2009). Adrenal function testing in pediatric cancer survivors. Pediatr Blood Cancer.

[51] Petraroli M, D’Alessio E, Ausili E, Barini A, Caradonna P, Riccardi R, Caldarelli M, Rossodivita A (2007). Bone mineral density in survivors of childhood brain tumours. Childs Nerv Syst.

[52] Mithal NP, Almond MK, Evans K, Hoskin PJ (1993). Reduced bone mineral density in long-term survivors of medulloblastoma. Br J Radiol.

[53] Krishnamoorthy P, Freeman C, Bernstein ML, Lawrence S, Rodd C (2004). Osteopenia in children who have undergone posterior fossa or craniospinal irradiation for brain tumors. Arch Pediatr Adolesc Med.

